# Approximate Counting of Minimal Unsatisfiable Subsets

**DOI:** 10.1007/978-3-030-53288-8_21

**Published:** 2020-06-13

**Authors:** Jaroslav Bendík, Kuldeep S. Meel

**Affiliations:** 8 Microsoft Research Lab, Redmond, WA USA; 9 University of Southern California, Los Angeles, CA USA; 10 Masaryk University, Brno, Czech Republic; 11 National University of Singapore, Singapore, Singapore

## Abstract

Given an unsatisfiable formula *F* in CNF, i.e. a set of clauses, the problem of Minimal Unsatisfiable Subset (MUS) seeks to identify a minimal subset of clauses $N \subseteq F$ such that *N* is unsatisfiable. The emerging viewpoint of MUSes as the root causes of unsatisfiability has led MUSes to find applications in a wide variety of diagnostic approaches. Recent advances in identification and enumeration of MUSes have motivated researchers to discover applications that can benefit from rich information about the set of MUSes. One such extension is that of counting the number of MUSes. The current best approach for MUS counting is to employ a MUS enumeration algorithm, which often does not scale for the cases with a reasonably large number of MUSes.

Motivated by the success of hashing-based techniques in the context of model counting, we design the first approximate MUS counting procedure with $(\varepsilon ,\delta )$ guarantees, called $\mathsf {AMUSIC}$. Our approach avoids exhaustive MUS enumeration by combining the classical technique of universal hashing with advances in QBF solvers along with a novel usage of union and intersection of MUSes to achieve runtime efficiency. Our prototype implementation of $\mathsf {AMUSIC}$ is shown to scale to instances that were clearly beyond the realm of enumeration-based approaches.

## Introduction

Given an unsatisfiable Boolean formula *F* as a set of clauses $\{f_1, f_2, \ldots f_n \}$, also known as conjunctive normal form (CNF), a set *N* of clauses is a Minimal Unsatisfiable Subset (MUS) of *F* iff $N \subseteq F$, *N* is unsatisfiable, and for each $f \in N$ the set $N \setminus \{f\}$ is satisfiable. Since MUSes can be viewed as representing the *minimal reasons* for unsatisfiability of a formula, MUSes have found applications in wide variety of domains ranging from diagnosis [45], ontologies debugging [1], spreadsheet debugging [29], formal equivalence checking [20], constrained counting and sampling [28], and the like. As the scalable techniques for identification of MUSes appeared only about decade and half ago, the earliest applications primarily focused on a reduction to the identification of a single MUS or a small set of MUSes. With an improvement in the scalability of MUS identification techniques, researchers have now sought to investigate extensions of MUSes and their corresponding applications. One such extension is MUS counting, i.e., counting the number of MUSes of *F*. Hunter and Konieczny [26], Mu [45], and Thimm [56] have shown that the number of MUSes can be used to compute different inconsistency metrics for general propositional knowledge bases.

In contrast to the progress in the design of efficient MUS identification techniques, the work on MUS counting is still in its nascent stages. Reminiscent of the early days of model counting, the current approach for MUS counting is to employ a complete MUS enumeration algorithm, e.g., [3, 12, 34, 55], to explicitly identify all MUSes. As noted in Sect. 2, there can be up to exponentially many MUSes of *F* w.r.t. |*F*|, and thus their complete enumeration can be practically intractable. Indeed, contemporary MUS enumeration algorithms often cannot complete the enumeration within a reasonable time [10, 12, 34, 47]. In this context, one wonders: *whether it is possible to design a scalable MUS counter without performing explicit enumeration of MUSes?*

The primary contribution of this paper is a probabilistic counter, called $\mathsf {AMUSIC}$, that takes in a formula *F*, tolerance parameter $\varepsilon$, confidence parameter $\delta$, and returns an estimate guaranteed to be within $(1+\varepsilon )$-multiplicative factor of the exact count with confidence at least $1-\delta$. Crucially, for *F* defined over *n* clauses, $\mathsf {AMUSIC}$ explicitly identifies only $\mathcal {O}(\log n \cdot \log (1/\delta )\cdot (\varepsilon )^{-2})$ many MUSes even though the number of MUSes can be exponential in *n*.

The design of $\mathsf {AMUSIC}$ is inspired by recent successes in the design of efficient XOR hashing-based techniques [15, 17] for the problem of model counting, i.e., given a Boolean formula *G*, compute the number of models (also known as solutions) of *G*. We observe that both the problems are defined over a power-set structure. In MUS counting, the goal is to count MUSes in the power-set of *F*, whereas in model counting, the goal is to count models in the power-set that represents all valuations of variables of *G*. Chakraborty et al. [18, 52] proposed an algorithm, called $\mathsf {ApproxMC}$, for approximate model counting that also provides the ($\epsilon$, $\delta$) guarantees. $\mathsf {ApproxMC}$ is currently in its third version, $\mathsf {ApproxMC3}$ [52]. The base idea of $\mathsf {ApproxMC3}$ is to partition the power-set into *nCells* small cells, then pick one of the cells, and count the number *inCell* of models in the cell. The total model count is then estimated as $nCells \times inCell$. Our algorithm for MUS counting is based on $\mathsf {ApproxMC3}$. We adopt the high-level idea to partition the power-set of *F* into small cells and then estimate the total MUS count based on a MUS count in a single cell. The difference between $\mathsf {ApproxMC3}$ and $\mathsf {AMUSIC}$ lies in the way of counting the target elements (models vs. MUSes) in a single cell; we propose novel MUS specific techniques to deal with this task. In particular, our contribution is the following:

- We introduce a QBF (quantified Boolean formula) encoding for the problem of counting MUSes in a single cell and use a $\varSigma _3^P$ oracle to solve it.
- Let $\mathtt {UMU}_F$ and $\mathtt {IMU}_F$ be the union and the intersection of all MUSes of *F*, respectively. We observe that every MUS of *F* (1) contains $\mathtt {IMU}_F$ and (2) is contained in $\mathtt {UMU}_F$. Consequently, if we determine the sets $\mathtt {UMU}_F$ and $\mathtt {IMU}_F$, then we can significantly speed up the identification of MUSes in a cell.
- We propose a novel approaches for computing the union $\mathtt {UMU}_F$ and the intersection $\mathtt {IMU}_F$ of all MUSes of *F*.
- We implement $\mathsf {AMUSIC}$ and conduct an extensive empirical evaluation on a set of *scalable* benchmarks. We observe that $\mathsf {AMUSIC}$ is able to compute estimates for problems clearly beyond the reach of existing enumeration-based techniques. We experimentally evaluate the *accuracy* of $\mathsf {AMUSIC}$. In particular, we observe that the estimates computed by $\mathsf {AMUSIC}$ are significantly closer to true count than the theoretical guarantees provided by $\mathsf {AMUSIC}$.

Our work opens up several new interesting avenues of research. From a theoretical perspective, we make polynomially many calls to a $\varSigma _3^P$ oracle while the problem of finding a MUS is known to be in $FP^{NP}$, i.e. a MUS can be found in polynomial time by executing a polynomial number of calls to an NP-oracle [19, 39]. Contrasting this to model counting techniques, where approximate counter makes polynomially many calls to an NP-oracle when the underlying problem of finding satisfying assignment is NP-complete, a natural question is to close the gap and seek to design a MUS counting algorithm with polynomially many invocations of an $FP^{NP}$ oracle. From a practitioner perspective, our work calls for a design of MUS techniques with native support for XORs; the pursuit of native support for XOR in the context of SAT solvers have led to an exciting line of work over the past decade [52, 53].

## Preliminaries and Problem Formulation

A Boolean formula $F = \{f_1, f_2, \ldots , f_n\}$ in a conjunctive normal form (CNF) is a set of Boolean clauses over a set of Boolean variables $Vars(F)$. A Boolean clause is a set $\{l_1, l_2, \ldots , l_k \}$ of literals. A literal is either a variable $x \in Vars(F)$ or its negation $\lnot x$. A truth assignment *I* to the variables $Vars(F)$ is a mapping $Vars(F) \rightarrow \{1, 0 \}$. A clause $f \in F$ is satisfied by an assignment *I* iff $I(l) = 1$ for some $l \in f$ or $I(k) = 0$ for some $\lnot k \in f$. The formula *F* is satisfied by *I* iff *I* satisfies every $f \in F$; in such a case *I* is called a *model* of *F*. Finally, *F* is *satisfiable* if it has a model; otherwise *F* is *unsatisfiable*.

A QBF is a Boolean formula where each variable is either universally ($\forall$) or existentially ($\exists$) quantified. We write $Q_1 \cdots Q_k$-QBF, where $Q_1, \ldots Q_k \in \{\forall , \exists \}$, to denote the class of QBF with a particular type of alternation of the quantifiers, e.g., $\exists \forall$-QBF or $\exists \forall \exists$-QBF. Every QBF is either true (valid) or false (invalid). The problem of deciding validity of a formula in $Q_1 \cdots Q_k$-QBF where $Q_1 = \exists$ is $\varSigma _k^P$-complete [43].

When it is clear from the context, we write just *formula* to denote either a QBF or a Boolean formula in CNF. Moreover, throughout the whole text, we use *F* to denote the input Boolean Formula in CNF. Furthermore, we will use capital letters, e.g., *S*, *K*, *N*, to denote other CNF formulas, small letters, e.g., $f, f_1, f_i$, to denote clauses, and small letters, e.g., $x,x',y$, to denote variables.

Given a set *X*, we write $$\mathcal {P}(X)$$ to denote the power-set of *X*, and |*X*| to denote the cardinality of *X*. Finally, we write $$ Pr [O : \mathbb {P}]$$ to denote the probability of an outcome *O* when sampling from a probability space $$\mathbb {P}$$. When $$\mathbb {P}$$ is clear from the context, we write just $$ Pr [O]$$.

**Minimal Unsatisfiability**

### Definition 1 (MUS)

A set *N*, $N \subseteq F$, is a *minimal unsatisfiable subset (MUS)* of *F* iff *N* is unsatisfiable and for all $f \in N$ the set $N \setminus \{f\}$ is satisfiable.

Note that the minimality concept used here is set minimality, not minimum cardinality. Therefore, there can be MUSes with different cardinalities. In general, there can be up to exponentially many MUSes of *F* w.r.t. |*F*| (see the Sperner’s theorem [54]). We use $\mathtt {AMU}_F$ to denote the set of all MUSes of *F*. Furthermore, we write $\mathtt {UMU}_F$ and $\mathtt {IMU}_F$ to denote the union and the intersection of all MUSes of F, respectively. Finally, note that every subset *S* of *F* can be expressed as a bit-vector over the alphabet $\{0,1\}$; for example, if $F = \{f_1, f_2, f_3, f_4 \}$ and $S = \{f_1, f_4 \}$, then the bit-vector representation of *S* is 1001.

### Definition 2

Let *N* be an unsatisfiable subset of *F* and $f \in N$. The clause *f* is *necessary* for *N* iff $N \setminus \{f\}$ is satisfiable.

The necessary clauses are sometimes also called *transition* [6] or *critical* [2] clauses. Note that a set *N* is a MUS iff every $f \in N$ is necessary for *N*. Also, note that a clause $f \in F$ is necessary for *F* iff $f \in \mathtt {IMU}_F$.

### Example 1

**Fig. 1.**
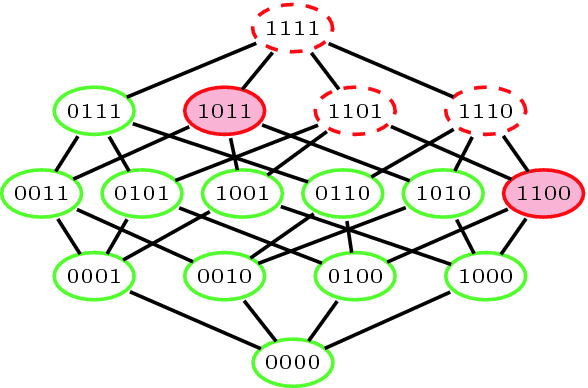
Illustration of the power set of the formula *F* from the Example 1. We denote individual subsets of *F* using the bit-vector representation. The subsets with a dashed border are the unsatisfiable subsets, and the others are satisfiable subsets. The MUSes are filled with a background color. (Color figure online)

We demonstrate the concepts on an example, illustrated in Fig. 1. Assume that $F = \{f_1 = \{x_1\}, f_2 = \{\lnot x_1\}, f_3 = \{x_2\}, f_4 = \{\lnot x_1, \lnot x_2\}\}$. In this case, $\mathtt {AMU}_F = \{\{f_1,f_2\}$, $\{f_1,f_3,f_4\}\}$, $\mathtt {IMU}_F = \{f_1\}$, and $\mathtt {UMU}_F = F$.

**Hash Functions**

Let *n* and *m* be positive integers such that $m < n$. By $\{1,0\}^n$ we denote the set of all bit-vectors of length *n* over the alphabet $\{1,0\}$. Given a vector $v \in \{1,0\}^n$ and $i \in \{1, \ldots , n\}$, we write *v*[*i*] to denote the *i*-th bit of *v*. A hash function *h* from a family $H_{xor}(n,m)$ of hash functions maps $\{1,0\}^n$ to $\{1,0\}^m$. The family $H_{xor}(n,m)$ is defined as $\{h\, |\, h(y)[i] = a_{i,0} \oplus (\bigoplus _{k = 1}^n (a_{i,k} \wedge y[k]))\, for\, all \, 1 \le i \le m\}$, where $\oplus$ and $\wedge$ denote the Boolean XOR and AND operators, respectively, and $a_{i,k} \in \{1,0\}$ for all $1 \le i \le m$ and $1 \le k \le n$.

To choose a hash function uniformly at random from $H_{xor}(n,m)$, we randomly and independently choose the values of $a_{i,k}$. It has been shown [24] that the family $H_{xor}(n,m)$ is pairwise independent, also known as strongly 2-universal. In particular, let us by $h \leftarrow H_{xor}(n,m)$ denote the probability space obtained by choosing a hash function *h* uniformly at random from $H_{xor}(n,m)$. The property of pairwise independence guarantees that for all $\alpha _1, \alpha _2 \in \{1,0\}^m$ and for all distinct $y_1, y_2 \in \{1,0\}^n$, $Pr [\bigwedge _{i = 1}^2 h(y_i) = \alpha _i : h \leftarrow H_{xor}(n,m)] = 2^{-2m}$.

We say that a hash function $h \in H_{xor}(n,m)$ *partitions* $\{ 0, 1 \}^n$ into $2^m$ *cells*. Furthermore, given a hash function $h \in H_{xor}(n,m)$ and a cell $\alpha \in \{1,0\}^m$ of *h*, we define their *prefix-slices*. In particular, for every $k \in \{1, \ldots , m\}$, the $k^{th}$ *prefix* of *h*, denoted $h^{(k)}$, is a map from $\{1,0\}^n$ to $\{1,0\}^k$ such that $h^{(k)}(y)[i] = h(y)[i]$ for all $y \in \{1,0\}^n$ and for all $i \in \{1,\ldots , k\}$. Similarly, the $k^{th}$ prefix of $\alpha$, denoted $\alpha ^{(k)}$, is an element of $\{1,0\}^k$ such that $\alpha ^{(k)}[i] = \alpha [i]$ for all $i \in \{1,\ldots , k\}$. Intuitively, a cell $\alpha ^{(k)}$ of $h^{(k)}$ originates by merging the two cells of $h^{(k + 1)}$ that differ only in the last bit.

In our work, we use hash functions from the family $$H_{xor}(n,m)$$ to partition the power-set $$\mathcal {P}(F)$$ of the given Boolean formula *F* into $$2^m$$ cells. Furthermore, given a cell $$\alpha \in \{ 0, 1 \}^m$$, let us by $$\mathtt {AMU}_{\langle F,h,\alpha \rangle }$$ denote the set of all MUSes in the cell $$\alpha $$; formally, $$ \mathtt {AMU}_{\langle F,h,\alpha \rangle } = \{ M \in \mathtt {AMU}_F\, | \, h( bit (M)) = \alpha \} $$, where $$ bit (M)$$ is the bit-vector representation of *M*. The following observation is crucial for our work.

### Observation 1

For every formula *F*, $m \in \{1, \ldots , |F|-1\}$, $h \in H_{xor}(|F|,m)$, and $\alpha \in \{0, 1 \}^{m}$ it holds that: $\mathtt {AMU}_{\langle F,h^{(i)},\alpha ^{(i)} \rangle } \supseteq \mathtt {AMU}_{\langle F,h^{(j)},\alpha ^{(j)} \rangle }$ for every $i < j$.

### Example 2

Assume that we are given a formula *F* such that $|F| = 4$ and a hash function $h \in H_{xor}(4,2)$ that is defined via the following values of individual $a_{i,k}$:

$$ \begin{array}{lllll} a_{1,0} = 0, &{}\quad \! a_{1,1} = 1, &{}\quad \! a_{1,2} = 1, &{}\quad \! a_{1,3} = 0, &{}\quad \! a_{1,4} = 1\\ a_{2,0} = 0, &{} \quad \! a_{2,1} = 1, &{}\quad \! a_{2,2} = 0, &{}\quad \! a_{2,3} = 0, &{}\quad \! a_{2,4} = 1\\ \end{array} $$

**Fig. 2.**
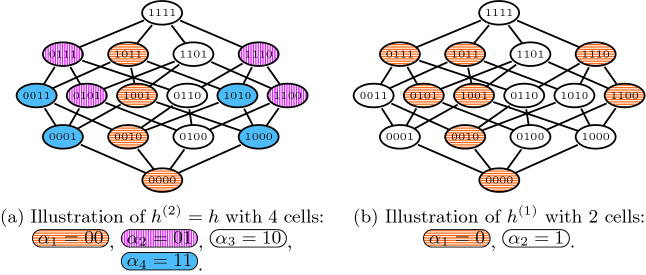
Illustration of the partition of $\mathcal {P}(F)$ by $h = h^{(2)}$ and $h^{(1)}$ from Example 2. In the case of *h*, we use 4 colors, orange, pink, white, and blue, to highlight its four cells. In case of $h^{(1)}$, there are only two cells: the white and the blue cells are merged into a white cell, and the pink and the orange cells are merged into an orange cell. (Color figure online)

The hash function partitions $\mathcal {P}(F)$ into 4 cells. For example, $h(1100) = 01$ since $h(1100)[1] = 0 \oplus (1\wedge 1) \oplus (1\wedge 1) \oplus (0\wedge 0) \oplus (1\wedge 0) = 0$ and $h(1100)[2] = 0 \oplus (1\wedge 1) \oplus (0\wedge 1) \oplus (0\wedge 0) \oplus (1\wedge 0) = 1$. Figure 2 illustrates the whole partition and also illustrates the partition given by the prefix $h^{(1)}$ of *h*.

### Problem Definitions

In this paper, we are concerned with the following problems.

**Name:**  problem

**Input:** A formula *F*, a tolerance $\epsilon > 0$, and a confidence $1 - \delta \in (0,1]$.

**Output:** A number *c* such that $Pr [|\mathtt {AMU}_F| / (1 + \epsilon ) \le c \le |\mathtt {AMU}_F|\cdot (1 + \epsilon ) ] \ge 1 - \delta$.

**Name:** MUS-membership problem

**Input:** A formula *F* and a clause $f \in F$.

**Output:** *True* if there is a MUS $M \in \mathtt {AMU}_F$ such that $f \in M$ and *False* otherwise.

**Name:** MUS-union problem

**Input:** A formula *F*.

**Output:** The union $\mathtt {UMU}_F$ of all MUSes of *F*.

**Name:** MUS-intersection problem

**Input:** A formula *F*.

**Output:** The intersection $\mathtt {IMU}_F$ of all MUSes of *F*.

**Name:**  problem

**Input:** A formula *F*, a tolerance $\epsilon > 0$, and a confidence $1 - \delta \in (0,1]$.

**Output:** A number *m* such that $Pr [m / (1 + \epsilon ) \le c \le m \cdot (1 + \epsilon )] \ge 1 - \delta$, where *m* is the number of models of *F*.

The main goal of this paper is to provide a solution to the  problem. We also deal with the MUS-membership, MUS-union and MUS-intersection problems since these problems emerge in our approach for solving the  problem. Finally, we do not focus on solving the  problem, however the problem is closely related to the  problem.

## Related Work

It is well-known (see e.g., [21, 36, 51]) that a clause $f \in F$ belongs to $\mathtt {IMU}_F$ iff *f* is necessary for *F*. Therefore, to compute $\mathtt {IMU}_F$, one can simply check each $f \in F$ for being necessary for *F*. We are not aware of any work that has focused on the MUS-intersection problem in more detail.

The MUS-union problem was recently investigated by Mencia et al. [42]. Their algorithm is based on gradually refining an *under*-approximation of $\mathtt {UMU}_F$ until the exact $\mathtt {UMU}_F$ is computed. Unfortunately, the authors experimentally show that their algorithm often fails to find the exact $\mathtt {UMU}_F$ within a reasonable time even for relatively small input instances (only an under-approximation is computed). In our work, we propose an approach that works in the other way: we start with an *over-approximation* of $\mathtt {UMU}_F$ and gradually refine the approximation to eventually get $\mathtt {UMU}_F$. Another related research was conducted by Janota and Marques-Silva [30] who proposed several QBF encodings for solving the MUS-membership problem. Although they did not focus on finding $\mathtt {UMU}_F$, one can clearly identify $\mathtt {UMU}_F$ by solving the MUS-membership problem for each $f \in F$.

As for counting the number of MUSes of *F*, we are not aware of any previous work dedicated to this problem. Yet, there have been proposed plenty of algorithms and tools (e.g., [3, 9, 11, 12, 35, 47]) for enumerating/identifying all MUSes of *F*. Clearly, if we enumerate all MUSes of *F*, then we obtain the exact value of $|\mathtt {AMU}_F|$, and thus we also solve the  problem. However, since there can be up to exponentially many of MUSes w.r.t. |*F*|, MUS enumeration algorithms are often not able to complete the enumeration in a reasonable time and thus are not able to find the value of $|\mathtt {AMU}_F|$.

Very similar to the  problem is the  problem. Both problems involve the same probabilistic and approximation guarantees. Moreover, both problems are defined over a power-set structure. In MUS counting, the goal is to count MUSes in $\mathcal {P}(F)$, whereas in model counting, the goal is to count models in $\mathcal {P}( Vars (F))$. In this paper, we propose an algorithm for solving the  problem that is based on $\mathsf {ApproxMC3}$ [15, 17, 52]. In particular, we keep the high-level idea of $\mathsf {ApproxMC3}$ for processing/exploring the power-set structure, and we propose new low-level techniques that are specific for MUS counting.

## $\mathsf {AMUSIC}$: A Hashing-Based MUS Counter

We now describe $\mathsf {AMUSIC}$, a hashing-based algorithm designed to solve the ($\varepsilon , \delta )$-#MUS problem. The name of the algorithm is an acronym for Approximate Minimal Unsatisfiable Subsets Implicit Counter. $\mathsf {AMUSIC}$ is based on $\mathsf {ApproxMC3}$, which is a hashing-based algorithm to solve $(\varepsilon ,\delta )$-#SAT problem. As such, while the high-level structure of $\mathsf {AMUSIC}$ and $\mathsf {ApproxMC3}$ share close similarities, the two algorithms differ significantly in the design of core technical subroutines.

We first discuss the high-level structure of $\mathsf {AMUSIC}$ in Sect. 4.1. We then present the key technical contributions of this paper: the design of core subroutines of $\mathsf {AMUSIC}$ in Sects. 4.3, 4.4 and 4.5.

### Algorithmic Overview

The main procedure of $\mathsf {AMUSIC}$ is presented in Algorithm 1. The algorithm takes as an input a Boolean formula *F* in CNF, a tolerance $\epsilon \,(> 0)$, and a confidence parameter $\delta \in (0,1]$, and returns an estimate of $|\mathtt {AMU}_F|$ within tolerance $\epsilon$ and with confidence at least $1 - \delta$. Similar to $\mathsf {ApproxMC3}$, we first check whether $|\mathtt {AMU}_F|$ is smaller than a specific $\mathsf {threshold}$ that is a function of $\varepsilon$. This check is carried out via a MUS enumeration algorithm, denoted $\mathsf {FindMUSes}$, that returns a set *Y* of MUSes of *F* such that $|Y| = \mathtt {min}(\mathsf {threshold}, |\mathtt {AMU}_F|)$. If $|Y| < \mathsf {threshold}$, the algorithm terminates while identifying the exact value of $|\mathtt {AMU}_F|$. In a significant departure from $\mathsf {ApproxMC3}$, $\mathsf {AMUSIC}$ subsequently computes the union ($\mathtt {UMU}_F$) and the intersection ($\mathtt {IMU}_F$) of all MUSes of *F* by invoking the subroutines $\mathsf {GetUMU}$ and $\mathsf {GetIMU}$, respectively. Through the lens of set representation of the CNF formulas, we can view $\mathtt {UMU}_F$ as another CNF formula, *G*. Our key observation is that $\mathtt {AMU}_F = \mathtt {AMU}_{G}$ (see Sect. 4.2), thus instead of working with the whole *F*, we can focus only on *G*. The rest of the main procedure is similar to $\mathsf {ApproxMC3}$, i.e., we repeatedly invoke the core subroutine called $\mathsf {AMUSICCore}$. The subroutine attempts to find an estimate *c* of $|\mathtt {AMU}_G|$ within the tolerance $\epsilon$. Briefly, to find the estimate, the subroutine partitions $\mathcal {P}(G)$ into $\mathsf {nCells}$ cells, then picks one of the cells, and counts the number $\mathsf {nSols}$ of MUSes in the cell. The pair $(\mathsf {nCells}, \mathsf {nSols})$ is returned by $\mathsf {AMUSICCore}$, and the estimate *c* of $|\mathtt {AMU}_G|$ is then computed as $\mathsf {nSols}\times \mathsf {nCells}$. There is a small chance that $\mathsf {AMUSICCore}$ fails to find the estimate; it such a case $\mathsf {nCells}= \mathsf {nSols}= \mathtt {null}$. Individual estimates are stored in a list *C*. After the final invocation of $\mathsf {AMUSICCore}$, $\mathsf {AMUSIC}$ computes the median of the list *C* and returns the median as the final estimate of $|\mathtt {AMU}_G|$. The total number of invocations of $\mathsf {AMUSICCore}$ is in $\mathcal {O}(\log (1/\delta ))$ which is enough to ensure the required confidence $1 - \delta$ (details on assurance of the $(\epsilon , \delta )$ guarantees are provided in Sect. 4.2). 
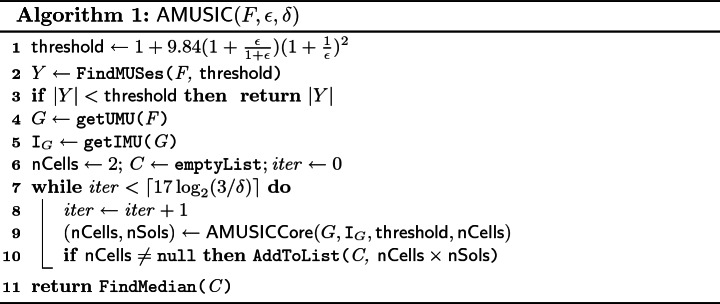
 
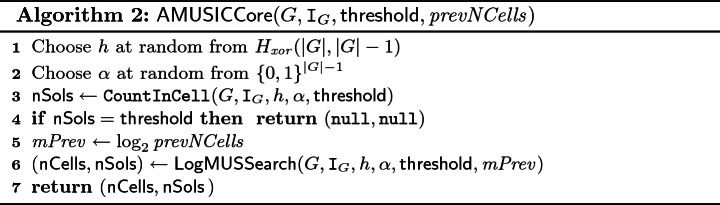


We now turn to $\mathsf {AMUSICCore}$ which is described in Algorithm 2. The partition of $\mathcal {P}(G)$ into $\mathsf {nCells}$ cells is made via a hash function *h* from $H_{xor}(|G|,m)$, i.e. $\mathsf {nCells}= 2^m$. The choice of *m* is a crucial part of the algorithm as it regulates the size of the cells. Intuitively, it is easier to identify all MUSes of a small cell; however, on the contrary, the use of small cells does not allow to achieve a reasonable tolerance. Based on $\mathsf {ApproxMC3}$, we choose *m* such that a cell given by a hash function $h \in H_{xor}(|G|,m)$ contains almost $\mathsf {threshold}$ many MUSes. In particular, the computation of $\mathsf {AMUSICCore}$ starts by choosing at random a hash function *h* from $H_{xor}(|G|,|G|-1)$ and a cell $\alpha$ at random from $\{0,1\}^{|G|-1}$. Subsequently, the algorithm tends to identify $m^{th}$ prefixes $h^{(m)}$ and $\alpha ^{(m)}$ of *h* and $\alpha$, respectively, such that $|\mathtt {AMU}_{\langle G,h^{(m)},\alpha ^{(m)}\rangle }| < \mathsf {threshold}$ and $|\mathtt {AMU}_{\langle G,h^{(m-1)},\alpha ^{(m-1)}\rangle }| \ge \mathsf {threshold}$. Recall that $\mathtt {AMU}_{\langle G,h^{(1)},\alpha ^{(1)}\rangle } \supseteq \cdots \supseteq \mathtt {AMU}_{\langle G,h^{(|G|-1)},\alpha ^{(|G|-1)}\rangle }$ (Observation 1, Sect. 2). We also know that the cell $\alpha ^{(0)}$, i.e. the whole $\mathcal {P}(G)$, contains at least $\mathsf {threshold}$ MUSes (see Algorithm 1, line 3). Consequently, there can exist at most one such *m*, and it exists if and only if $|\mathtt {AMU}_{\langle G,h^{(|G|-1)},\alpha ^{(|G|-1)}\rangle }| < \mathsf {threshold}$. Therefore, the algorithm first checks whether $|\mathtt {AMU}_{\langle G,h^{(|G|-1)},\alpha ^{(|G|-1)}\rangle }| < \mathsf {threshold}$. The check is carried via a procedure $\mathsf {CountInCell}$ that returns the number $\mathsf {nSols}= \mathtt {min}(|\mathtt {AMU}_{\langle G,h^{(|G|-1)},\alpha ^{(|G|-1)}\rangle }|,\mathsf {threshold})$. If $\mathsf {nSols}= \mathsf {threshold}$, then $\mathsf {AMUSICCore}$ fails to find the estimate of $|\mathtt {AMU}_G|$ and terminates. Otherwise, a procedure $\mathsf {LogMUSSearch}$ is used to find the required value of *m* together with the number $\mathsf {nSols}$ of MUSes in $\alpha ^{(m)}$. The implementation of $\mathsf {LogMUSSearch}$ is directly adopted from $\mathsf {ApproxMC3}$ and thus we do not provide its pseudocode here (note that in $\mathsf {ApproxMC3}$ the procedure is called $\mathsf {LogSATSearch}$). We only briefly summarize two main ingredients of the procedure. First, it has been observed that the required value of *m* is often similar for repeated calls of $\mathsf {AMUSICCore}$. Therefore, the algorithm keeps the value *mPrev* of *m* from previous iteration and first test values near *mPrev*. If none of the near values is the required one, the algorithm exploits that $\mathtt {AMU}_{\langle G,h^{(1)},\alpha ^{(1)}\rangle } \supseteq \cdots \supseteq \mathtt {AMU}_{\langle G,h^{(|G|-1)},\alpha ^{(|G|-1)}\rangle }$, which allows it to find the required value of *m* via the galloping search (variation of binary search) while performing only $\log |G|$ calls of $\mathsf {CountInCell}$.

Note that in $\mathsf {ApproxMC3}$, the procedure $\mathsf {CountInCell}$ is called $\mathsf {BSAT}$ and it is implemented via an NP oracle, whereas we use a $\varSigma _3^P$ oracle to implement the procedure (see Sect. 4.3). The high-level functionality is the same: the procedures use up to $\mathsf {threshold}$ calls of the oracle to check whether the number of the target elements (models vs. MUSes) in a cell is lower than $\mathsf {threshold}$.

### Analysis and Comparison with $\mathsf {ApproxMC3}$

Following from the discussion above, there are three crucial technical differences between $\mathsf {AMUSIC}$ and $\mathsf {ApproxMC3}$: (1) the implementation of the subroutine $\mathsf {CountInCell}$ in the context of MUS, (2) computation of the intersection $\mathtt {IMU}_F$ of all MUSes of *F* and its usage in $\mathsf {CountInCell}$, and (3) computation of the union $\mathtt {UMU}_F$ of all MUSes of *F* and invocation of the underlying subroutines with *G* (i.e., $\mathtt {UMU}_F$) instead of *F*. The usage of $\mathsf {CountInCell}$ can be viewed as domain-specific instantiation of $\mathsf {BSAT}$ in the context of MUSes. Furthermore, we use the computed intersection of MUSes to improve the runtime efficiency of $\mathsf {CountInCell}$. It is perhaps worth mentioning that prior studies have observed that over 99% of the runtime of $\mathsf {ApproxMC3}$ is spent inside the subroutine $\mathsf {BSAT}$ [52]. Therefore, the runtime efficiency of $\mathsf {CountInCell}$ is crucial for the runtime performance of $\mathsf {AMUSIC}$, and we discuss in detail, in Sect. 4.3, algorithmic contributions in the context of $\mathsf {CountInCell}$ including usage of $\mathtt {IMU}_F$. We now argue that the replacement of *F* with *G* in line 4 in Algorithm 1 does not affect correctness guarantees, which is stated formally below:

#### Lemma 1

For every $G'$ such that $\mathtt {UMU}_{F} \subseteq G' \subseteq F$, the following hold:

1 $$\begin{aligned} \mathtt {AMU}_F = \mathtt {AMU}_{G'} \end{aligned}$$

2 $$\begin{aligned} \mathtt {IMU}_F = \mathtt {IMU}_{G'} \end{aligned}$$

#### Proof

(1) Since $G' \subseteq F$ then every MUS of $G'$ is also a MUS of *F*. In the other direction, every MUS of *F* is contained in the union $\mathtt {UMU}_F$ of all MUSes of *F*, and thus every MUS of *F* is also a MUS of $G'$ ($\supseteq \mathtt {UMU}_{F}$).

(2) $$\mathtt {IMU}_F = \bigcap _{M \in \mathtt {AMU}_F} = \bigcap _{M \in \mathtt {AMU}_{G'}} = \mathtt {IMU}_{G'}$$.

Equipped with Lemma 1, we now argue that each run of $\mathsf {AMUSIC}$ can be simulated by a run of $\mathsf {ApproxMC3}$ for an appropriately chosen formula. Given an unsatisfiable formula $F = \{f_1, \ldots , f_{|F|}\}$, let us by $B_F$ denote a satisfiable formula such that: (1) $Vars (B_F) = \{x_1, \ldots , x_{|F|}\}$ and (2) an assignment $I: Vars (B_F) \rightarrow \{1,0\}$ is a model of $B_F$ iff $\{f_i | I(x_i) = 1\}$ is a MUS of *F*. Informally, models of $B_F$ one-to-one map to MUSes of *F*. Hence, the size of sets returned by $\mathsf {CountInCell}$ for *F* is identical to the corresponding $\mathsf {BSAT}$ for $B_F$. Since the analysis of $\mathsf {ApproxMC3}$ only depends on the correctness of the size of the set returned by $\mathsf {BSAT}$, we conclude that the answer computed by $\mathsf {AMUSIC}$ would satisfy $(\varepsilon ,\delta )$ guarantees. Furthermore, observing that $\mathsf {CountInCell}$ makes $\mathsf {threshold}$ many queries to $\varSigma _3^P$-oracle, we can bound the time complexity. Formally,

#### Theorem 1

Given a formula *F*, a tolerance $\varepsilon > 0$, and a confidence $1 - \delta \in (0, 1]$, let $\mathsf {AMUSIC}(F,\varepsilon ,\delta )$ return *c*. Then $Pr [|\mathtt {AMU}_F| / (1 + \epsilon ) \le c \le |\mathtt {AMU}_F|\cdot (1 + \epsilon ) ] \ge 1 - \delta$. Furthermore, $\mathsf {AMUSIC}$ makes $\mathcal {O}(\log |F| \cdot \frac{1}{\varepsilon ^2} \cdot \log (1/\delta ))$ calls to $\varSigma _3^P$ oracle.

Few words are in order concerning the complexity of $$\mathsf {AMUSIC}$$. As noted in Sect. 1, for a formula on *n* variables, approximate model counters make $$\mathcal {O}(\log n \cdot \frac{1}{\varepsilon ^2} \cdot \log (1/\delta ))$$ calls to an NP oracle, whereas the complexity of finding a satisfying assignment is NP-complete. In our case, we make calls to a $$\varSigma _3^P$$ oracle while the problem of finding a MUS is in $$ FP ^{ NP }$$. Therefore, a natural direction of future work is to investigate the design of a hashing-based technique that employs an $$ FP ^{ NP }$$ oracle.

### Counting MUSes in a Cell: $\mathsf {CountInCell}$


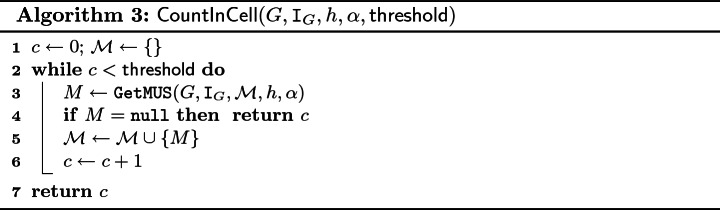
 In this section, we describe the procedure CountInCell. The input of the procedure is the formula *G* (i.e., $\mathtt {UMU}_F$), the set $\mathtt {I}_G = \mathtt {IMU}_G$, a hash function $h \in H_{xor}(|G|, m)$, a cell $\alpha \in \{0,1\}^m$, and the $\mathsf {threshold}$ value. The output is $c = min (\mathsf {threshold}, |\mathtt {AMU}_{\langle G,h,\alpha \rangle }|)$.

The description is provided in Algorithm 3. The algorithm iteratively calls a procedure GetMUS that returns either a MUS *M* such that $M \in (\mathtt {AMU}_{\langle G,h,\alpha \rangle } \setminus \mathcal {M})$ or null if there is no such MUS. For each *M*, the value of *c* is increased and *M* is added to $\mathcal {M}$. The loop terminates either when *c* reaches the value of $\mathsf {threshold}$ or when GetMUS fails to find a new MUS (i.e., returns null). Finally, the algorithm returns *c*.

**GetMUS.** To implement the procedure GetMUS, we build an $\exists \forall \exists$-QBF formula MUSInCell such that each witness of the formula corresponds to a MUS from $\mathtt {AMU}_{\langle G,h,\alpha \rangle } \setminus \mathcal {M}$. The formula consists of several parts and uses several sets of variables that are described in the following.

The main part of the formula, shown in Eq. (3), introduces the first existential quantifier and a set $$P = \{p_1, \ldots , p_{|G|} \}$$ of variables that are quantified by the quantifier. Note that each valuation *I* of *P* corresponds to a subset *S* of *G*; in particular let us by $$I_{P,G}$$ denote the set $$\{ f_i \in G \, | \, I(p_i) = 1 \}$$. The formula is build in such a way that a valuation *I* is a witness of the formula if and only if $$I_{P,G}$$ is a MUS from $$\mathtt {AMU}_{\langle G,h,\alpha \rangle } \setminus \mathcal {M}$$. This property is expressed via three conjuncts, denoted  ,  , and  , encoding that (i) $$I_{P,G}$$ is in the cell $$\alpha $$, (ii) $$I_{P,G}$$ is not in $$\mathcal {M}$$, and (iii) $$I_{P,G}$$ is a MUS, respectively.3Recall that the family $$H_{xor}(n,m)$$ of hash functions is defined as $$\{h\, |\, h(y)[i] = a_{i,0} \oplus (\bigoplus _{k = 1}^n a_{i,k} \wedge y[k])\, for\, all \, 1 \le i \le m\}$$, where $$a_{i,k} \in \{0,1\}$$ (Sect. 2). A hash function $$h \in H_{xor}(n,m)$$ is given by fixing the values of individual $$a_{i,k}$$ and a cell $$\alpha $$ of *h* is a bit-vector from $$\{0,1\}^m$$. The formula $$\mathtt {inCell}(P)$$ encoding that the set $$I_{P,G}$$ is in the cell $$\alpha $$ of *h* is shown in Eq. (4).4To encode that we are not interested in MUSes from $$\mathcal {M}$$, we can simply block all the valuations of *P* that correspond to these MUSes. However, we can do better. In particular, recall that if *M* is a MUS, then no proper subset and no proper superset of *M* can be a MUS; thus, we prune away all these sets from the search space. The corresponding formula is shown in Eq. (5).5The formula $$\mathtt {isMUS}(P)$$ encoding that $$I_{P,G}$$ is a MUS is shown in Eq. (6). Recall that $$I_{P,G}$$ is a MUS if and only if $$I_{P,G}$$ is unsatisfiable and for every *closest subset* *S* of $$I_{P,G}$$ it holds that *S* is satisfiable, where *closest subset* means that $$|I_{P,G} \setminus S| = 1$$. We encode these two conditions using two subformulas denoted by $$\mathtt {unsat}(P)$$ and $$\mathtt {noUnsatSubset}(P)$$.6The formula  , shown in Eq. (7), introduces the set $$ Vars (G)$$ of variables that appear in *G* and states that every valuation of $$ Vars (G)$$ falsifies at least one clause contained in $$I_{P,G}$$.7The formula  , shown in Eq. (8), introduces another set of variables: $$Q = \{q_1, \ldots , q_{|G|} \}$$. Similarly as in the case of *P*, each valuation *I* of *Q* corresponds to a subset of *G* defined as $$I_{Q,G} = \{ f_i \in G \, | \, I(q_i) = 1\}$$. The formula expresses that for every valuation *I* of *Q* it holds that $$I_{Q,G}$$ is satisfiable or $$I_{Q,G}$$ is not a closest subset of $$I_{P,G}$$.8The requirement that $$I_{Q,G}$$ is satisfiable is encoded in Eq. (9). Since we are already reasoning about the satisfiability of *G*’s clauses in Eq. (7), we introduce here a copy $$G'$$ of *G* where each variable $$x_i$$ of *G* is substituted by its primed copy $$x_i'$$. Equation (9) states that there exists a valuation of $$ Vars (G')$$ that satisfies $$I_{Q,G}$$.9Equation (10) encodes that $$I_{Q,G}$$ is a closest subset of $$I_{P,G}$$. To ensure that $$I_{Q,G}$$ is a *subset* of $$I_{P,G}$$, we add the clauses $$q_i \rightarrow p_i$$. To ensure the *closeness*, we use cardinality constraints. In particular, we introduce another set $$R = \{r_1, \ldots , r_{|G|} \}$$ of variables and enforce their values via $$r_i \leftrightarrow (p_i \wedge \lnot q_i)$$. Intuitively, the number of variables from *R* that are set to 1 equals to $$|I_{P,G} \setminus I_{Q,G}|$$. Finally, we add cardinality constraints, denoted by  , ensuring that exactly one $$r_i$$ is set to 1.10Note that instead of encoding a *closest subset* in Eq. 10, we could just encode that $$I_{Q,G}$$ is an arbitrary proper subset of $$I_{P,G}$$ as it would still preserve the meaning of Eq. 6 that $$I_{P,G}$$ is a MUS. Such an encoding would not require introducing the set *R* of variables and also, at the first glance, would save a use of one existential quantifier. The thing is that the whole formula would still be in the form of $$\exists \forall \exists $$-QBF due to Eq. 9 (which introduces the second existential quantifier). The advantage of using a closet subset is that we significantly prune the search space of the QBF solver. It is thus matter of contemporary QBF solvers whether it is more beneficial to reduce the number of variables (by removing *R*) or to prune the searchspace via *R*.

For the sake of lucidity, we have not exploited the knowledge of $\mathtt {IMU}_G$ ($I_G$) while presenting the above equations. Since we know that every clause $f \in \mathtt {IMU}_G$ has to be contained in every MUS of *G*, we can fix the values of the variables $\{ p_i \, | \, f_i \in \mathtt {IMU}_G \}$ to 1. This, in turn, significantly simplifies the equations and prunes away exponentially many (w.r.t. $|\mathtt {IMU}_G|$) valuations of *P*, *Q*, and *R*, that need to be assumed. To solve the final formula, we employ a $\exists \forall \exists$-QBF solver, i.e., a $\varSigma _3^P$ oracle.

Finally, one might wonder why we use our custom solution for identifying MUSes in a cell instead of employing one of existing MUS extraction techniques. Conventional MUS extraction algorithms cannot be used to identify MUSes that are in a cell since the cell is not “continuous” w.r.t. the set containment. In particular, assume that we have three sets of clauses, *K*, *L*, *M*, such that $K \subset L \subset M$. It can be the case that *K* and *M* are in the cell, but *L* is not in the cell. Contemporary MUS extraction techniques require the search space to be continuous w.r.t. the set containment and thus cannot be used in our case.

### Computing $\mathtt {UMU}_F$

We now turn our attention to computing the union $\mathtt {UMU}_F$ (i.e., *G*) of all MUSes of *F*. Let us start by describing well-known concepts of *autark variables* and a *lean kernel*. A set $A \subseteq Vars (F)$ of variables is an *autark* of *F* iff there exists a truth assignment to *A* such that every clause of *F* that contains a variable from *A* is satisfied by the assignment [44]. It holds that the union of two autark sets is also an autark set, thus there exists a unique largest autark set (see, e.g., [31, 32]). The *lean kernel* of *F* is the set of all clauses that do not contain any variable from the largest autark set. It is known that the *lean kernel* of *F* is an over-approximation of $\mathtt {UMU}_F$ (see e.g., [31, 32]), and there were proposed several algorithms, e.g., [33, 38], for computing the lean kernel.

**Algorithm.** Our approach for computing $\mathtt {UMU}_F$ consists of two parts. First, we compute the lean kernel *K* of *F* to get an over-approximation of $\mathtt {UMU}_F$, and then we gradually refine the over-approximation *K* until *K* is exactly the set $\mathtt {UMU}_F$. The refinement is done by solving the MUS-membership problem for each $f \in K$. To solve the MUS-membership problem efficiently, we reveal a connection to necessary clauses, as stated in the following lemma.

#### Lemma 2

A clause $f \in F$ belongs to $\mathtt {UMU}_F$ iff there is a subset *W* of *F* such that *W* is unsatisfiable and *f* is necessary for *W* (i.e., $W \setminus \{f\}$ is satisfiable).

#### Proof

$\Rightarrow :$ Let $f \in \mathtt {UMU}_F$ and $M \in \mathtt {AMU}_F$ such that $f \in M$. Since *M* is a MUS then $M \setminus \{f\}$ is satisfiable; thus *f* is necessary for *M*.

$\Leftarrow :$ If *W* is a subset of *F* and $f \in W$ a necessary clause for *W* then *f* has to be contained in every MUS of *W*. Moreover, *W* has at least one MUS and since $W \subseteq F$, then every MUS of *W* is also a MUS of *F*.

Our approach for computing $\mathtt {UMU}_F$ is shown in Algorithm 4. It takes as an input the formula *F* and outputs $\mathtt {UMU}_F$ (denoted *K*). Moreover, the algorithm maintains a set $\mathcal {M}$ of MUSes of *F*. Initially, $\mathcal {M} = \emptyset$ and *K* is set to the lean kernel of *F*; we use an approach by Marques-Silva et al. [38] to compute the lean kernel. At this point, we know that $K \supseteq \mathtt {UMU}_F \supseteq \{f \in M\, |\, M \in \mathcal {M} \}$. To find $\mathtt {UMU}_F$, the algorithm iteratively determines for each $f \in K \setminus \{f \in M\, |\, M \in \mathcal {M} \}$ if $f \in \mathtt {UMU}_F$. In particular, for each *f*, the algorithm checks whether there exists a subset *W* of *K* such that *f* is necessary for *W* (Lemma 2). The task of finding *W* is carried out by a procedure  . If there is no such *W*, then the algorithm removes *f* from *K*. In the other case, if *W* exists, the algorithm finds a MUS of *W* and adds the MUS to the set $\mathcal {M}$. Any available single MUS extraction approach, e.g., [2, 5, 7, 46], can be used to find the MUS.
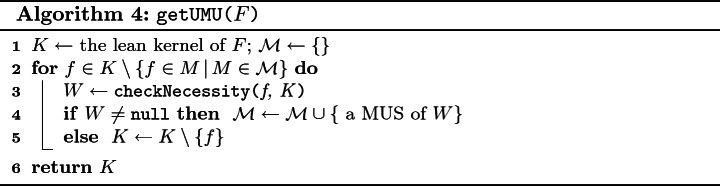


To implement the procedure  we build a QBF formula that is true iff there exists a set $W \subseteq K$ such that *W* is unsatisfiable and *f* is necessary for *W*. To represent *W* we introduce a set $S = \{s_g \, |\, g \in K \}$ of Boolean variables; each valuation *I* of *S* corresponds to a subset $I_{S,K}$ of *K* defined as $I_{S,K} = \{ g \in K\, |\, I(s_g) = 1 \}$. Our encoding is shown in Eq. 11.

11 $$\begin{aligned} \exists S, Vars (K).\, \forall Vars (K').\, s_f \wedge (\bigwedge _{g \in K \setminus \{f\}} (g \vee \lnot s_g)) \wedge ( \bigvee _{g \in K'} (\lnot g \wedge s_g)) \end{aligned}$$

The formula consists of three main conjuncts. The first conjunct ensures that *f* is present in $$I_{S,K}$$. The second conjunct states that $$I_{S,K} \setminus \{f\}$$ is satisfiable, i.e., that there exists a valuation of $$ Vars (K)$$ that satisfies $$I_{S,K} \setminus \{f\}$$. Finally, the last conjunct express that $$I_{S,K} $$ is unsatisfiable, i.e., that every valuation of $$ Vars (K)$$ falsifies at least one clause of $$I_{S,K}$$. Since we are already reasoning about variables of *K* in the second conjunct, in the third conjunct, we use a primed version (a copy) $$K'$$ of *K*.

**Alternative QBF Encodings.** Janota and Marques-Silva [30] proposed three other QBF encodings for the MUS-membership problem, i.e., for deciding whether a given $f \in F$ belongs to $\mathtt {UMU}_F$. Two of the three proposed encodings are typically inefficient; thus, we focus on the third encoding, which is the most concise among the three. The encoding, referred to as JM encoding (after the initials of the authors), uses only two quantifiers in the form of $\exists \forall$-QBF and it is only linear in size w.r.t. |*F*|. The underlying ideas by JM encoding and our encoding differ significantly. Our encoding is based on necessary clauses (Lemma 2), whereas JM exploits a connection to so-called *Maximal Satisfiable Subsets*. Both the encodings use the same quantifiers; however, our encoding is smaller. In particular, the JM uses $2 \times ( Vars (F) + |F|)$ variables whereas our encoding uses only $|F| + 2 \times Vars (F)$ variables, and leads to smaller formulas.

**Implementation.** Recall that we compute $\mathtt {UMU}_F$ to reduce the search space, i.e. instead of working with the whole *F*, we work only with $G = \mathtt {UMU}_F$. The soundness of this reduction is witnessed in Lemma 1 (Sect. 4.2). In fact, Lemma 1 shows that it is sound to reduce the search space to any $G'$ such that $\mathtt {UMU}_F \subseteq G' \subseteq F$. Since our algorithm for computing $\mathtt {UMU}_F$ subsumes repeatedly solving a $\varSigma _2^{P}$-complete problem, it can be very time-consuming. Therefore, instead of computing the exact $\mathtt {UMU}_F$, we optionally compute only an over-approximation $G'$ of $\mathtt {UMU}_F$. In particular, we set a (user-defined) time limit for computing the lean kernel *K* of *F*. Moreover, we use a time limit for executing the procedure  ; if the time limit is exceeded for a clause $f \in K$, we conservatively assume that $f \in \mathtt {UMU}_F$, i.e., we over-approximate.

**Sparse Hashing and** $\mathtt {UMU}_F$. The approach of computation of $\mathtt {UMU}_F$ is similar to, in spirit, computation of independent support of a formula to design sparse hash functions [16, 28]. Briefly, given a Boolean formula *H*, an *independent support* of *H* is a set $\mathcal {I} \subseteq Vars (H)$ such that in every model of *H*, the truth assignment to $\mathcal {I}$ uniquely determines the truth assignment to $Vars (H) \setminus \mathcal {I}$. Practically, independent support can be used to reduce the search space where a model counting algorithm searches for models of *H*. It is interesting to note that the state of the art technique reduces the computation of independent support of a formula in the context of model counting to that of computing (Group) Minimal Unsatisfiable Subset (GMUS). Thus, a formal study of computation of independent support in the context of MUSes is an interesting direction of future work.

### Computing $\mathtt {IMU}_G$


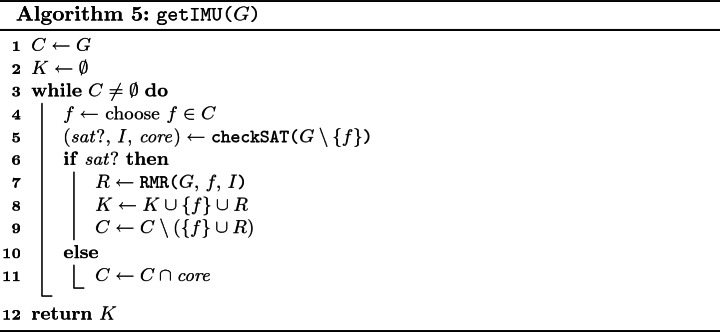


Our approach to compute the intersection $\mathtt {IMU}_G$ (i.e., $I_G$) of all MUSes of *G* is composed of several ingredients. First, recall that a clause $f \in G$ belongs to $\mathtt {IMU}_G$ iff *f* is necessary for *G*. Another ingredient is the ability of contemporary SAT solvers to provide either a model or an *unsat core* of a given unsatisfiable formula $N \subseteq G$, i.e., a small, yet not necessarily minimal, unsatisfiable subset of *N*. The final ingredient is a technique called *model rotation*. The technique was originally proposed by Marques-Silva and Lynce [40], and it serves to explore necessary clauses based on other already known necessary clauses. In particular, let *f* be a necessary clause for *G* and $I: Vars (G) \rightarrow \{0,1\}$ a model of $G \setminus \{f\}$. Since *G* is unsatisfiable, the model *I* does not satisfy *f*. The model rotation attempts to alter *I* by switching, one by one, the Boolean assignment to the variables $Vars (\{f\})$. Each variable assignment $I'$ that originates from such an alternation of *I* necessarily satisfies *f* and does not satisfy at least one $f' \in G$. If it is the case that there is exactly one such $f'$, then $f'$ is necessary for *G*. An improved version of model rotation, called *recursive model rotation*, was later proposed by Belov and Marques-Silva [6] who noted that the model rotation could be recursively performed on the newly identified necessary clauses.

Our approach for computing $\mathtt {IMU}_G$ is shown in Algorithm 5. To find $\mathtt {IMU}_G$, the algorithm decides for each *f* whether *f* is necessary for *G*. In particular, the algorithm maintains two sets: a set *C* of *candidates* on necessary clauses and a set *K* of already known necessary clauses. Initially, *K* is empty and $C = G$. At the end of computation, *C* is empty and *K* equals to $\mathtt {IMU}_G$. The algorithm works iteratively. In each iteration, the algorithm picks a clause $f \in C$ and checks $G \setminus \{f\}$ for satisfiability via a procedure $\mathtt {checkSAT}$. Moreover, $\mathtt {checkSAT}$ returns either a model *I* or an unsat core $core$ of $G \setminus \{f\}$. If $G \setminus \{f\}$ is satisfiable, i.e. *f* is necessary for *G*, the algorithm employs the recursive model rotation, denoted by  , to identify a set *R* of additional necessary clauses. Subsequently, all the newly identified necessary clauses are added to *K* and removed from *C*. In the other case, when $G \setminus \{f\}$ is unsatisfiable, the set *C* is reduced to $C \cap core$ since every necessary clause of *G* has to be contained in every unsatisfiable subset of *G*. Note that $f \not \in core$, thus at least one clause is removed from *C*.

## Experimental Evaluation

We employed several external tools to implement $\mathsf {AMUSIC}$. In particular, we use the QBF solver CAQE [49] for solving the QBF formula MUSInCell, the 2QBF solver CADET [50] for solving our $\exists \forall$-QBF encoding while computing $\mathtt {UMU}_F$, and the QBF preprocessor QRATPre+ [37] for preprocessing/simplifying our QBF encodings. Moreover, we employ muser2 [7] for a single MUS extraction while computing $\mathtt {UMU}_F$, a MaxSAT solver UWrMaxSat [48] to implement the algorithm by Marques-Silva et al. [38] for computing the lean kernel of *F*, and finally, we use a toolkit called pysat [27] for encoding cardinality constraints used in the formula MUSInCell. The tool along with all benchmarks that we used is available at https://github.com/jar-ben/amusic.

**Objectives.** As noted earlier, $\mathsf {AMUSIC}$ is the first technique to (approximately) count MUSes without explicit enumeration. We demonstrate the efficacy of our approach via a comparison with two state of the art techniques for MUS enumeration: $\mathsf {MARCO}$ [35] and $\mathsf {MCSMUS}$ [3]. Within a given time limit, a MUS enumeration algorithm either identifies the whole $\mathtt {AMU}_F$, i.e., provides the exact value of $|\mathtt {AMU}_F|$, or identifies just a subset of $\mathtt {AMU}_F$, i.e., provides an under-approximation of $|\mathtt {AMU}_F|$ with no approximation guarantees.

The objective of our empirical evaluation was two-fold: First, we experimentally examine the scalability of $\mathsf {AMUSIC}$, $\mathsf {MARCO}$, and $\mathsf {MCSMUS}$ w.r.t. $|\mathtt {AMU}_F|$. Second, we examine the *empirical accuracy* of $\mathsf {AMUSIC}$.

**Benchmarks and Experimental Setup.** Given the lack of dedicated counting techniques, there is no sufficiently large set of publicly available benchmarks to perform critical analysis of counting techniques. To this end, we focused on a recently emerging theme of evaluation of SAT-related techniques on *scalable benchmarks*^1^. In keeping with prior studies employing empirical methodology based on scalable benchmarks [22, 41], we generated a custom collection of CNF benchmarks. The benchmarks mimic requirements on multiprocessing systems. Assume that we are given a system with two groups (kinds) of processes, $A = \{a_1, \ldots , a_{|A|} \}$ and $B = \{b_1, \ldots , b_{|B|} \}$, such that $|A| \ge |B|$. The processes require resources of the system; however, the resources are limited. Therefore, there are restrictions on which processes can be active simultaneously. In particular, we have the following three types of mutually independent restrictions on the system:

- The first type of restriction states that “at most $k - 1$ processes from the group *A* can be active simultaneously”, where $k \le |A|$.
- The second type of restriction enforces that “if no process from *B* is active then at most $k-1$ processes from *A* can be active, and if at least one process from *B* is active then at most $l - 1$ processes from *A* can be active”, where $k,l \le |A|$.
- The third type of restriction includes the second restriction. Moreover, we assume that a process from *B* can activate a process from *A*. In particular, for every $b_i \in B$, we assume that when $b_i$ is active, then $a_i$ is also active.

**Fig. 3.**
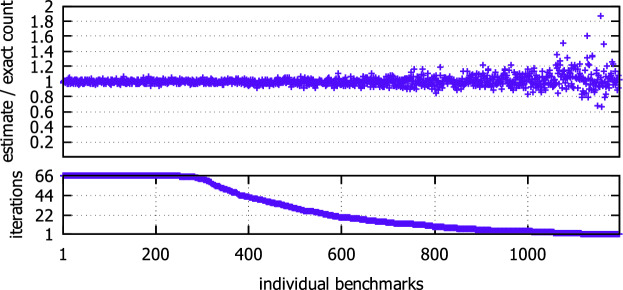
The number of completed iterations and the accuracy of the final MUS count estimate for individual benchmarks.

We encode the three restrictions via three Boolean CNF formulas, $R_1$, $R_2$, $R_3$. The formulas use three sets of variables: $X = \{x_1, \ldots , x_{|A|}\}$, $Y = \{y_1, \ldots , y_{|B|}\}$, and *Z*. The sets *X* and *Y* represent the Boolean information about activity of processes from *A* and *B*: $a_i$ is active iff $x_i = 1$ and $b_j$ is active iff $y_j = 1$. The set *Z* contains additional auxiliary variables. Moreover, we introduce a formula $\mathtt {ACT} = (\bigwedge _{x_i \in X} x_i) \wedge (\bigwedge _{y_i \in Y} y_i)$ encoding that all processes are active. For each $i \in \{1,2,3\}$, the conjunction $G_i = R_i \wedge \mathtt {ACT}$ is unsatisfiable. Intuitively, every MUS of $G_i$ represents a minimal subset of processes that need to be active to violate the restriction. The number of MUSes in $G_1$, $G_2$, and $G_3$ is ${|A| \atopwithdelims ()k}$, ${|A| \atopwithdelims ()k} + |B|\times {|A| \atopwithdelims ()l}$, and ${|A| \atopwithdelims ()k} + \sum _{i=1}^{|B|} ({|B| \atopwithdelims ()i} \times {|A| - 1 \atopwithdelims ()l - i})$, respectively. We generated $G_1$, $G_2$, and $G_3$ for these values: $10 \le |A| \le 30$, $2 \le |B| \le 6$, ${\lfloor }{\frac{|A|}{2}}{\rfloor } \le k \le {\lfloor }{\frac{3\times |A|}{2}}{\rfloor }$, and $l = k - 1$. In total, we obtained 1353 benchmarks (formulas) that range in their size from 78 to 361 clauses, use from 40 to 152 variables, and contain from 120 to $1.7 \times 10^9$ MUSes.

All experiments were run using a time limit of 7200 s and computed on an AMD EPYC 7371 16-Core Processor, 1 TB memory machine running Debian Linux 4.19.67-2. The values of $\epsilon$ and $\delta$ were set to 0.8 and 0.2, respectively.

**Accuracy.** Recall that to compute an estimate *c* of $|\mathtt {AMU}_F|$, $\mathsf {AMUSIC}$ performs multiple iteration of executing $\mathsf {AMUSICCore}$ to get a list *C* of multiple estimates of $|\mathtt {AMU}_F|$, and then use the median of *C* as the final estimate *c*. The more iterations are performed, the higher is the confidence that *c* is within the required tolerance $\epsilon = 0.8$, i.e., that $\frac{|\mathtt {AMU}_F|}{1.8} \le c \le 1.8\cdot |\mathtt {AMU}_F|$. To achieve the confidence $1 - \delta = 0.8$, 66 iterations need to be performed. In case of 157 benchmarks, the algorithm was not able to finish even a single iteration, and only in case of 251 benchmarks, the algorithm finished all the 66 iterations. For the remaining 945 benchmarks, at least some iterations were finished, and thus at least an estimate with a lower confidence was determined.

We illustrate the achieved results in Fig. 3. The figure consists of two plots. The plot at the bottom of the figure shows the number of finished iterations (y-axis) for individual benchmarks (x-axis). The plot at the top of the figure shows how accurate were the MUS count estimates. In particular, for each benchmark (formula) *F*, we show the number $\frac{c}{|\mathtt {AMU}_F|}$ where *c* is the final estimate (median of estimates from finished iterations). For benchmarks where all iterations were completed, it was always the case that the final estimate is within the required tolerance, although we had only 0.8 theoretical confidence that it would be the case. Moreover, the achieved estimate never exceeded a tolerance of 0.1, which is much better than the required tolerance of 0.8. As for the benchmarks where only some iterations were completed, there is only a single benchmark where the tolerance of 0.8 was exceeded.

**Fig. 4.**
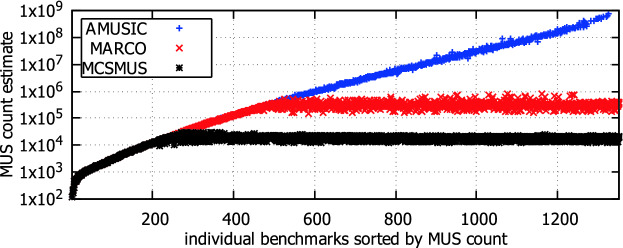
Scalability of $\mathsf {AMUSIC}$, $\mathsf {MARCO}$, and $\mathsf {MCSMUS}$ w.r.t. $|\mathtt {AMU}_F|$.

**Scalability.** The scalability of $\mathsf {AMUSIC}$, $\mathsf {MARCO}$, and $\mathsf {MCSMUS}$ w.r.t. the number of MUSes ($|\mathtt {AMU}_F|$) is illustrated in Fig. 4. In particular, for each benchmark (x-axis), we show in the plot the estimate of the MUS count that was achieved by the algorithms (y-axis). The benchmarks are sorted by the exact count of MUSes in the benchmarks. $\mathsf {MARCO}$ and $\mathsf {MCSMUS}$ were able to finish the MUS enumeration, and thus to provide the count, only for benchmarks that contained at most $10^6$ and $10^5$ MUSes, respectively. $\mathsf {AMUSIC}$, on the other hand, was able to provide estimates on the MUS count even for benchmarks that contained up to $10^9$ MUSes. Moreover, as we have seen in Fig. 3, the estimates are very accurate. Only in the case of 157 benchmarks where $\mathsf {AMUSIC}$ finished no iteration, it could not provide any estimate.

## Summary and Future Work

We presented a probabilistic algorithm, called $\mathsf {AMUSIC}$, for approximate MUS counting that needs to explicitly identify only logarithmically many MUSes and yet still provides strong theoretical guarantees. The high-level idea is adopted from a model counting algorithm $\mathsf {ApproxMC3}$: we partition the search space into small cells, then count MUSes in a single cell, and estimate the total count by scaling the count from the cell. The novelty lies in the low-level algorithmic parts that are specific for MUSes. Mainly, (1) we propose QBF encoding for counting MUSes in a cell, (2) we exploit MUS intersection to speed-up localization of MUSes, and (3) we utilize MUS union to reduce the search space significantly. Our experimental evaluation showed that the scalability of $\mathsf {AMUSIC}$ outperforms the scalability of contemporary enumeration-based counters by several orders of magnitude. Moreover, the practical accuracy of $\mathsf {AMUSIC}$ is significantly better than what is guaranteed by the theoretical guarantees.

Our work opens up several questions at the intersection of theory and practice. From a theoretical perspective, the natural question is to ask if we can design a scalable algorithm that makes polynomially many calls to an ${ NP }$ oracle. From a practical perspective, our work showcases interesting applications of QBF solvers with native XOR support. Since approximate counting and sampling are known to be inter-reducible, another line of work would be to investigate the development of an almost-uniform sampler for MUSes, which can potentially benefit from the framework proposed in UniGen [14, 16]. Another line of work is to extend our MUS counting approach to other constraint domains where MUSes find an application, e.g., *F* can be a set of SMT [25] or LTL [4, 8] formulas or a set of transition predicates [13, 23].

## Footnotes

1 M. Y. Vardi, in his talk at BIRS CMO 18w5208 workshop, called on the SAT community to focus on scalable benchmarks in lieu of competition benchmarks. Also, see: https://gitlab.com/satisfiability/scalablesat (Accessed: May 10, 2020).
